# Supplementary material for: Transcriptome Analysis Reveals Vimentin-Induced Disruption of Cell–Cell Associations Augments Breast Cancer Cell Migration
Source: Cells. 2022 Dec 13;11(24):4035. doi: 10.3390/cells11244035 (PMC9776984; doi:10.3390/cells11244035)
Supplement: Supplementary file 1 [file cells-11-04035-s001.zip › DEGs and enrichment analysis.zip!/DEGs and enrichment analysis/WT_VIMvsVector KEGG/GSEA/WT_VIM/ARGININE_AND_PROLINE_METABOLISM/ARGININE_AND_PROLINE_METABOLISM(HSA00330).html]

Details for gene set ARGININE\_AND\_PROLINE\_METABOLISM(HSA00330)[GSEA]

|  || Dataset | fpkm.sample |
| Phenotype | sample.cls |
| Upregulated in class | WT\_VIM |
| GeneSet | ARGININE\_AND\_PROLINE\_METABOLISM(HSA00330) |
| Enrichment Score (ES) | 0.65861857 |
| Normalized Enrichment Score (NES) | 1.143734 |
| Nominal p-value | 0.0 |
| FDR q-value | 0.28924844 |
| FWER p-Value | 0.652 |
Table: GSEA Results Summary

  

Fig 1: Enrichment plot: ARGININE\_AND\_PROLINE\_METABOLISM(HSA00330)      
 Profile of the Running ES Score & Positions of GeneSet Members on the Rank Ordered List

  

| PROBE | DESCRIPTION (from dataset) | GENE SYMBOL | GENE\_TITLE | RANK IN GENE LIST | RANK METRIC SCORE | RUNNING ES | CORE ENRICHMENT || 1 | ENSG00000233719 | GOT2P3 |  |  | 176 | Infinity | -0.0029 | Yes |
| 2 | ENSG00000228339 | AMD1P1 |  |  | 400 | Infinity | -0.0066 | Yes |
| 3 | ENSG00000089250 | NOS1 |  |  | 1293 | Infinity | -0.0216 | Yes |
| 4 | ENSG00000260386 | LINC01225 |  |  | 1788 | Infinity | -0.0299 | Yes |
| 5 | ENSG00000104524 | PYCR3 |  |  | 2418 | 21.700 | 0.1551 | Yes |
| 6 | ENSG00000007171 | NOS2 |  |  | 2478 | 12.538 | 0.2671 | Yes |
| 7 | ENSG00000261096 | AC073476.3 |  |  | 2658 | 7.265 | 0.3296 | Yes |
| 8 | ENSG00000166165 | CKB |  |  | 3046 | 4.684 | 0.3653 | Yes |
| 9 | ENSG00000116649 | SRM |  |  | 3784 | 3.294 | 0.3825 | Yes |
| 10 | ENSG00000137124 | ALDH1B1 |  |  | 3798 | 3.278 | 0.4118 | Yes |
| 11 | ENSG00000130005 | GAMT |  |  | 3826 | 3.248 | 0.4406 | Yes |
| 12 | ENSG00000183010 | PYCR1 |  |  | 4577 | 2.627 | 0.4516 | Yes |
| 13 | ENSG00000069535 | MAOB |  |  | 4766 | 2.510 | 0.4711 | Yes |
| 14 | ENSG00000250799 | PRODH2 |  |  | 4846 | 2.470 | 0.4920 | Yes |
| 15 | ENSG00000161132 | AC007663.1 |  |  | 5518 | 2.192 | 0.5004 | Yes |
| 16 | ENSG00000172508 | CARNS1 |  |  | 6717 | 1.873 | 0.4970 | Yes |
| 17 | ENSG00000223572 | CKMT1A |  |  | 6728 | 1.869 | 0.5137 | Yes |
| 18 | ENSG00000277196 | AC007325.2 |  |  | 6854 | 1.852 | 0.5282 | Yes |
| 19 | ENSG00000116771 | AGMAT |  |  | 7806 | 1.657 | 0.5271 | Yes |
| 20 | ENSG00000120053 | GOT1 |  |  | 8029 | 1.619 | 0.5379 | Yes |
| 21 | ENSG00000115758 | ODC1 |  |  | 8585 | 1.537 | 0.5424 | Yes |
| 22 | ENSG00000232389 | AL583856.1 |  |  | 8778 | 1.511 | 0.5527 | Yes |
| 23 | ENSG00000159423 | ALDH4A1 |  |  | 9259 | 1.444 | 0.5576 | Yes |
| 24 | ENSG00000059573 | ALDH18A1 |  |  | 9346 | 1.437 | 0.5691 | Yes |
| 25 | ENSG00000237289 | CKMT1B |  |  | 9808 | 1.382 | 0.5738 | Yes |
| 26 | ENSG00000143149 | ALDH9A1 |  |  | 9830 | 1.379 | 0.5858 | Yes |
| 27 | ENSG00000241935 | HOGA1 |  |  | 9913 | 1.370 | 0.5968 | Yes |
| 28 | ENSG00000218793 | AL049697.3 |  |  | 10434 | 1.318 | 0.5999 | Yes |
| 29 | ENSG00000125166 | GOT2 |  |  | 10717 | 1.292 | 0.6067 | Yes |
| 30 | ENSG00000213080 | AL354714.2 |  |  | 10874 | 1.277 | 0.6156 | Yes |
| 31 | ENSG00000102172 | SMS |  |  | 10880 | 1.276 | 0.6270 | Yes |
| 32 | ENSG00000143811 | PYCR2 |  |  | 11008 | 1.264 | 0.6363 | Yes |
| 33 | ENSG00000072210 | ALDH3A2 |  |  | 11029 | 1.261 | 0.6473 | Yes |
| 34 | ENSG00000249779 | AC106800.2 |  |  | 11380 | 1.230 | 0.6525 | Yes |
| 35 | ENSG00000065154 | OAT |  |  | 11663 | 1.213 | 0.6586 | Yes |
| 36 | ENSG00000142920 | AZIN2 |  |  | 13352 | 1.076 | 0.6397 | No |
| 37 | ENSG00000131730 | CKMT2 |  |  | 13681 | 1.051 | 0.6436 | No |
| 38 | ENSG00000123505 | AMD1 |  |  | 13801 | 1.041 | 0.6510 | No |
| 39 | ENSG00000133313 | CNDP2 |  |  | 13932 | 1.032 | 0.6581 | No |
| 40 | ENSG00000002549 | LAP3 |  |  | 14666 | 0.991 | 0.6546 | No |
| 41 | ENSG00000171766 | GATM |  |  | 15374 | 0.948 | 0.6512 | No |
| 42 | ENSG00000081181 | ARG2 |  |  | 16308 | 0.899 | 0.6435 | No |
| 43 | ENSG00000072682 | P4HA2 |  |  | 17760 | 0.812 | 0.6262 | No |
| 44 | ENSG00000100033 | PRODH |  |  | 18378 | 0.777 | 0.6228 | No |
| 45 | ENSG00000244556 | ODCP |  |  | 19186 | 0.733 | 0.6157 | No |
| 46 | ENSG00000141504 | SAT2 |  |  | 19550 | 0.713 | 0.6160 | No |
| 47 | ENSG00000251400 | ALDH7A1P1 |  |  | 19617 | 0.709 | 0.6213 | No |
| 48 | ENSG00000002726 | AOC1 |  |  | 20509 | 0.660 | 0.6121 | No |
| 49 | ENSG00000164904 | ALDH7A1 |  |  | 21479 | 0.611 | 0.6012 | No |
| 50 | ENSG00000149380 | P4HA3 |  |  | 22286 | 0.558 | 0.5926 | No |
| 51 | ENSG00000150656 | CNDP1 |  |  | 23316 | 0.488 | 0.5795 | No |
| 52 | ENSG00000111275 | ALDH2 |  |  | 23631 | 0.468 | 0.5784 | No |
| 53 | ENSG00000189221 | MAOA |  |  | 24199 | 0.421 | 0.5726 | No |
| 54 | ENSG00000164867 | NOS3 |  |  | 24772 | 0.375 | 0.5663 | No |
| 55 | ENSG00000122884 | P4HA1 |  |  | 24862 | 0.366 | 0.5681 | No |
| 56 | ENSG00000249286 | AMD1P3 |  |  | 25260 | 0.324 | 0.5643 | No |
| 57 | ENSG00000118520 | ARG1 |  |  | 26099 | 0.240 | 0.5523 | No |
| 58 | ENSG00000130066 | SAT1 |  |  | 26351 | 0.209 | 0.5499 | No |
| 59 | ENSG00000230528 | NOS2P3 |  |  | 27318 | 0.000 | 0.5335 | No |
| 60 | ENSG00000216378 | AL080285.1 |  |  | 27525 | 0.000 | 0.5300 | No |
| 61 | ENSG00000279557 | AC010435.1 |  |  | 28349 | 0.000 | 0.5161 | No |
| 62 | ENSG00000215124 | AMD1P4 |  |  | 29228 | 0.000 | 0.5012 | No |
| 63 | ENSG00000104879 | CKM |  |  | 30529 | 0.000 | 0.4792 | No |
| 64 | ENSG00000263604 | AC015688.1 |  |  | 30871 | NaN | 0.4735 | No |
| 65 | ENSG00000260622 | AC073476.2 |  |  | 31090 | NaN | 0.4699 | No |
| 66 | ENSG00000234489 | ALDH7A1P4 |  |  | 32810 | NaN | 0.4409 | No |
| 67 | ENSG00000231900 | GOT2P1 |  |  | 33173 | NaN | 0.4348 | No |
| 68 | ENSG00000225448 | AC009477.1 |  |  | 33188 | NaN | 0.4347 | No |
| 69 | ENSG00000265788 | AC015688.4 |  |  | 33294 | NaN | 0.4330 | No |
| 70 | ENSG00000233634 | GOT2P5 |  |  | 33771 | NaN | 0.4250 | No |
| 71 | ENSG00000260889 | CKBP1 |  |  | 35724 | NaN | 0.3920 | No |
| 72 | ENSG00000213500 | LAP3P2 |  |  | 36136 | NaN | 0.3852 | No |
| 73 | ENSG00000237345 | AC245047.6 |  |  | 36791 | NaN | 0.3742 | No |
| 74 | ENSG00000226474 | AP000532.1 |  |  | 38026 | NaN | 0.3534 | No |
| 75 | ENSG00000229365 | AC006967.1 |  |  | 38076 | NaN | 0.3526 | No |
| 76 | ENSG00000234391 | AL606490.6 |  |  | 39987 | NaN | 0.3204 | No |
| 77 | ENSG00000214745 | AL391419.1 |  |  | 41116 | NaN | 0.3013 | No |
| 78 | ENSG00000249795 | AC097462.2 |  |  | 41683 | NaN | 0.2918 | No |
| 79 | ENSG00000281450 | PANDAR |  |  | 42932 | NaN | 0.2708 | No |
| 80 | ENSG00000257767 | AC002996.1 |  |  | 43674 | NaN | 0.2583 | No |
| 81 | ENSG00000237801 | AMD1P2 |  |  | 44279 | NaN | 0.2482 | No |
| 82 | ENSG00000234448 | AL606490.7 |  |  | 45352 | NaN | 0.2301 | No |
| 83 | ENSG00000271299 | AC008962.1 |  |  | 45601 | NaN | 0.2260 | No |
| 84 | ENSG00000257224 | CR383656.2 |  |  | 45846 | NaN | 0.2219 | No |
| 85 | ENSG00000232292 | ALDH7A1P2 |  |  | 46346 | NaN | 0.2136 | No |
| 86 | ENSG00000227616 | AC063976.3 |  |  | 48308 | NaN | 0.1804 | No |
| 87 | ENSG00000232392 | AC002366.1 |  |  | 48848 | NaN | 0.1714 | No |
| 88 | ENSG00000266647 | AC053481.4 |  |  | 50688 | NaN | 0.1403 | No |
| 89 | ENSG00000231489 | AL606490.4 |  |  | 50915 | NaN | 0.1366 | No |
| 90 | ENSG00000226971 | AL606490.2 |  |  | 51187 | NaN | 0.1321 | No |
| 91 | ENSG00000224656 | AL009172.1 |  |  | 52383 | NaN | 0.1119 | No |
| 92 | ENSG00000229173 | AC108059.1 |  |  | 52484 | NaN | 0.1103 | No |
| 93 | ENSG00000234792 | AL450023.2 |  |  | 52489 | NaN | 0.1104 | No |
| 94 | ENSG00000225397 | AC244505.3 |  |  | 53184 | NaN | 0.0987 | No |
| 95 | ENSG00000110887 | DAO |  |  | 53539 | NaN | 0.0928 | No |
| 96 | ENSG00000233139 | AC244636.2 |  |  | 54125 | NaN | 0.0830 | No |
| 97 | ENSG00000167494 | NOS2P2 |  |  | 54313 | NaN | 0.0799 | No |
| 98 | ENSG00000225055 | AL606490.1 |  |  | 54594 | NaN | 0.0752 | No |
| 99 | ENSG00000243853 | ALDH7A1P3 |  |  | 54725 | NaN | 0.0731 | No |
| 100 | ENSG00000231603 | AL606490.5 |  |  | 54772 | NaN | 0.0724 | No |
| 101 | ENSG00000230849 | GOT2P2 |  |  | 55251 | NaN | 0.0644 | No |
| 102 | ENSG00000223958 | AC244505.2 |  |  | 55712 | NaN | 0.0567 | No |
| 103 | ENSG00000215448 | SRMP1 |  |  | 55947 | NaN | 0.0528 | No |
| 104 | ENSG00000264892 | NOS2P4 |  |  | 55956 | NaN | 0.0528 | No |
| 105 | ENSG00000282738 | AC243829.7 |  |  | 56301 | NaN | 0.0471 | No |
| 106 | ENSG00000234780 | AC245047.5 |  |  | 56969 | NaN | 0.0358 | No |
| 107 | ENSG00000272917 | AC010186.4 |  |  | 57190 | NaN | 0.0322 | No |
| 108 | ENSG00000251342 | AC026774.2 |  |  | 57200 | NaN | 0.0321 | No |
| 109 | ENSG00000229826 | AC244505.5 |  |  | 57302 | NaN | 0.0305 | No |
| 110 | ENSG00000228354 | AL450023.1 |  |  | 58103 | NaN | 0.0171 | No |
| 111 | ENSG00000256450 | AC007552.1 |  |  | 58514 | NaN | 0.0102 | No |
| 112 | ENSG00000220091 | LAP3P1 |  |  | 58770 | NaN | 0.0060 | No |
| 113 | ENSG00000258485 | SRMP2 |  |  | 58823 | NaN | 0.0052 | No |
| 114 | ENSG00000229662 | AC245047.2 |  |  | 59089 | NaN | 0.0008 | No |
Table: GSEA details [plain text format]

  

Fig 2: ARGININE\_AND\_PROLINE\_METABOLISM(HSA00330)      
 Blue-Pink O' Gram in the Space of the Analyzed GeneSet

  

Fig 3: ARGININE\_AND\_PROLINE\_METABOLISM(HSA00330): Random ES distribution      
 Gene set null distribution of ES for **ARGININE\_AND\_PROLINE\_METABOLISM(HSA00330)**
